# Editorial: Myeloid cells as active players in human neurodegenerative diseases

**DOI:** 10.3389/fnins.2025.1712394

**Published:** 2025-10-22

**Authors:** Abhirami K. Iyer, Miguel Moutinho, Pinar Ayata, Hande Karahan

**Affiliations:** ^1^Department of Psychiatry, Washington University in St Louis, St Louis, MO, United States; ^2^Stark Neurosciences Research Institute, Indiana University School of Medicine, Indianapolis, IN, United States; ^3^Department of Medical and Molecular Genetics, Indiana University School of Medicine, Indianapolis, IN, United States; ^4^Neuroscience Initiative, Advanced Science Research Center, Graduate Program in Biology, Graduate Program in Biochemistry, The City University of New York (CUNY) Graduate Center, New York, NY, United States

**Keywords:** microglia, myeloid cells, Alzheimer's disease, Parkinson's disease, neurodegeneration, aging, biomarkers

An emerging theme across various neurodegenerative diseases is the crucial role of myeloid cells, including brain-resident microglia as well as perivascular and border-associated macrophages, in driving neuroinflammation and contributing to disease progression (Zhang et al., 2023; Heneka et al., 2025). This Research Topic brings together up-to-date reviews and original research on how these immune cells shape disease risk, progression, and modulate aging-related changes within the brain. Additionally, the collected work highlights the emerging potential of myeloid biomarkers as diagnostic or monitoring tools for neurodegenerative diseases.

Aging is a significant risk factor for all neurodegenerative diseases (Hou et al., 2019). In addition to neuronal changes, brain aging involves microglia-specific cellular processes, including impaired homeostatic functions, chronic inflammation, cellular dystrophy and senescence, as well as lipid droplet accumulation (Antignano et al., 2023). The original research article by Assale et al. investigates how *SIGLEC11*, a recently identified Alzheimer's disease (AD) risk gene (Bellenguez et al., 2022) and exclusively expressed in microglia, affects the functions of microglia and macrophages in the context of aging. The authors demonstrate that overexpression of *SIGLEC11* in aged mice reduces the number of activated microglia, as well as inflammatory and oxidative stress markers and lipid droplet accumulation. Importantly, *SIGLEC11* overexpression prevented the dopaminergic neuron loss in the substantia nigra that typically accompanies aging. This study identifies SIGLEC11 as a crucial microglia-specific brake on age-associated neuroinflammatory processes, highlighting that microglial immune checkpoints can be leveraged to promote neuronal survival and health. This research also opens the possibility of testing SIGLEC11 ligands as potential therapies to slow neurodegeneration in aging brains. While this research avenue is promising, further investigation is warranted for a deeper understanding of how SIGLEC11 interacts with other microglia-specific AD risk factors and key regulators, such as TREM2, MS4A4A, and CD33 (McQuade and Blurton-Jones, 2019), across different stages of aging and disease progression.

In addition to AD, there is a growing appreciation for the role of microglia in Parkinson's disease (PD) (Kam et al., 2020). The mini-review article by Trainor et al. presents evidence supporting microglia as both responders and contributors to PD pathogenesis and progression, in contrast to the historical perspective of microglia as secondary responders to the neurodegeneration process. The authors discuss the impact of PD risk genes (*SNCA, PRKN, PINK1, PARK7, GBA1, LRRK2, and VPS35*) on key microglial functions, including vesicular trafficking, lysosomal machinery, and mitochondrial function. While these processes have traditionally been studied from a neuron-centric perspective (Kam et al., 2020), the authors emphasize ongoing research efforts to delineate the diversity of cell types involved and the cellular functions that go awry. Cell-type-specific manipulation of disease risk genes, along with the broader use of disease-specific human stem cell-based models, is highlighted to gain a more comprehensive understanding of the mechanisms underlying neurodegeneration.

The immune system's involvement in neurodegeneration is not limited to microglia. Several emerging studies implicate a role of conventional (CD4+ and CD8+) and invariant (NKT and MAIT) T cells in neurological diseases (Wyatt-Johnson et al., 2024). However, very few studies exist on the *a priori* steps of antigen presentation, a requisite for T cell activation in the context of neurodegeneration. Afify et al. discuss the limited scientific literature investigating the role of classical (MHC class I and II) and non-classical (CD1d and MR1) antigen-presenting molecules in neurological diseases, such as AD and related dementias, Amyotrophic Lateral Sclerosis, Multiple Sclerosis (MS), and PD. The authors emphasize the importance of advancing this research area to better understand T cell involvement in neurodegeneration and to develop readily accessible, peripheral immune-centric therapeutic approaches aimed at modulating neuroinflammation.

Myeloid cells have garnered significant attention not only for their influence on disease pathogenesis but also for their potential as biomarkers for diagnosing or monitoring neurodegenerative diseases (Noh et al., 2025). Building on this perspective, Kodosaki et al. review the role of the myeloid branch of hematopoietic stem cells, including monocyte, macrophage, and dendritic cell lineages, granulocytes, such as neutrophils, erythrocytes/platelets, and the brain-resident innate immune sentinels—microglia, across three major neurodegenerative diseases: AD, PD, and MS. The authors highlight the potential of leveraging myeloid biomarkers as prognostic, diagnostic, or monitoring tools for these diseases. The review also highlights the gaps, challenges, and recent advances in methodologies to improve biofluid and imaging myeloid biomarker research for neurological diseases, emphasizing the variability within and across different neurodegenerative diseases.

From novel myeloid biomarkers and antigen presentation pathways to the impact of PD risk genes on microglia biology, and the striking discovery of SIGLEC11 as a crucial microglia receptor that limits neuroinflammation and neuronal loss during aging, these articles collectively highlight novel and promising research directions for diagnosis, therapy, and mechanistic understanding of neurodegenerative diseases. Together, they underscore the growing importance of studies focusing on myeloid cells to gain insights and ultimately modulate neurodegeneration.
